# Rapid Development of Pernicious Anemia Unmasking Underlying Gastric Adenocarcinoma

**DOI:** 10.7759/cureus.13630

**Published:** 2021-03-01

**Authors:** Vihitha Thota, Mahati Paravathaneni, Verushka Bedi, Michael Branham, Rajesh Thirumaran

**Affiliations:** 1 Internal Medicine, Mercy Catholic Medical Center, Darby, USA; 2 Internal Medicine, Drexel University College of Medicine, Philadelphia, USA; 3 Hematology/Oncology, Mercy Catholic Medical Center, Darby, USA

**Keywords:** gastric adenocarcinoma, pernicious anemia, b12 deficiency, intrinsic factor, folfox, macrocytic anemia

## Abstract

Gastric cancer is one of the most common malignancies, often detected at later stages as patients remain asymptomatic until later stages. Pernicious anemia (PA), a well-known cause of vitamin B12 deficiency, is a classic risk factor for gastric cancer. Patients with PA usually present with megaloblastic anemia and peripheral neuropathy; however, they can also present with nonspecific symptoms. We describe below the case of a 56-year-old male who presented with symptoms of nausea, vomiting, and fatigue. Initial lab work revealed severe B12 deficiency, pancytopenia, and intramedullary hemolysis warranting endoscopy that revealed a gastric fundus mass significant for adenocarcinoma on biopsy.

## Introduction

Gastric cancer is a prevalent, fatal malignancy with a high mortality and poor prognosis. Common causes of gastric cancer include *Helicobacter pylori* infection, tobacco smoking, heavy alcohol use, age, and diet. Pernicious anemia (PA), a well-recognized cause of vitamin B12 deficiency, is defined as autoimmune destruction of the intrinsic factor (IF) glycoprotein, or destruction of the gastric body and fundal parietal cells that produce IF [1]. PA remains a less common but a classic risk factor for primary gastric cancer. We present to you a case of a 56-year-old male diagnosed with severe vitamin B12 deficiency, with further workup unmasking underlying PA and gastric adenocarcinoma. Our case is unique in that it depicts the precipitous nature of the disease, with our patient developing B12 deficiency in a remarkably short period of time.

## Case presentation

A 56-year-old male with a past medical history of hypertension and hyperlipidemia presented to our hospital with a one-day history of nausea and nonbloody, nonbilious vomiting associated with fatigue for one week prior to presentation. He denied any abdominal pain, diarrhea, constipation, bleeding per rectum, sick contacts, or recent travel history. He reported having a screening colonoscopy five years before presentation, which had been benign; however, he never had an endoscopy done. On presentation, physical examination exhibited scleral icterus but was otherwise unremarkable, with a benign abdominal exam. Bloodwork was notable for hemoglobin (Hgb) of 4.7 g/dL with mean corpuscular volume (MCV) 124.2 fL, and a creatinine of 3.4 mg/dL (baseline 1.3 mg/dL). Interestingly, upon reviewing his outpatient lab work, the patient’s Hgb was 14.0 g/dL with MCV 97.3 fL a year prior.

**Figure 1:**
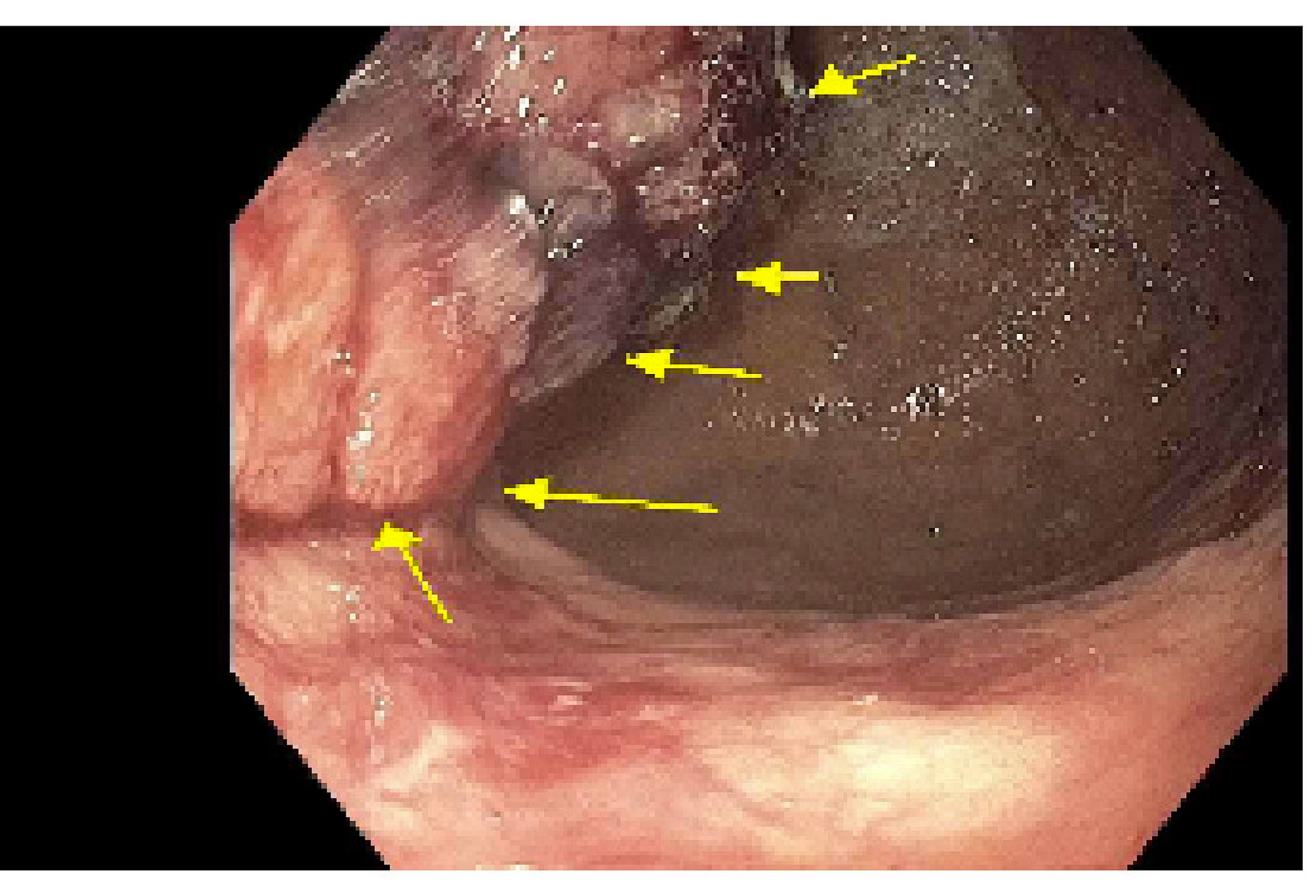
Mass within the gastric fundus as seen on endoscopy.

He was started on IV fluid hydration, IV pantoprazole twice a day, and transfused multiple units of packed red blood cells. Further workup revealed iron 120 ug/dL, total iron-binding capacity (TIBC) 264 mcg/dL, transferrin 211 mg/dL, and ferritin 760.4 ng/mL. Vitamin B12 level was undetectable at <50 pg/mL (reference 180-914 pg/mL) and red blood cell (RBC) folate was 977 ng/mL (reference 280-791 ng/mL). Lactate dehydrogenase (LDH) was elevated at 4922 U/L with haptoglobin undetectable at <30 mg/dL. Soon after admission, the patient developed pancytopenia with WBC 4.4 Thou/uL, Hgb 7.3 g/dL, and platelet count 119 Thou/uL, indicating intramedullary hemolysis, with schistocytes seen on the peripheral blood smear. He was started on intramuscular (IM) vitamin B12 supplementation with 1000 mcg daily for severe vitamin B12 deficiency. He eventually underwent endoscopy, which revealed mild gastritis and a large, sessile, partially circumferential (involving one-half of the lumen circumference) mass in the gastric fundus, with no active bleeding or stigmata of recent bleeding, with protrusion into the cardio-esophageal junction (CEJ) area (Figure 1).

**Figure 2:**
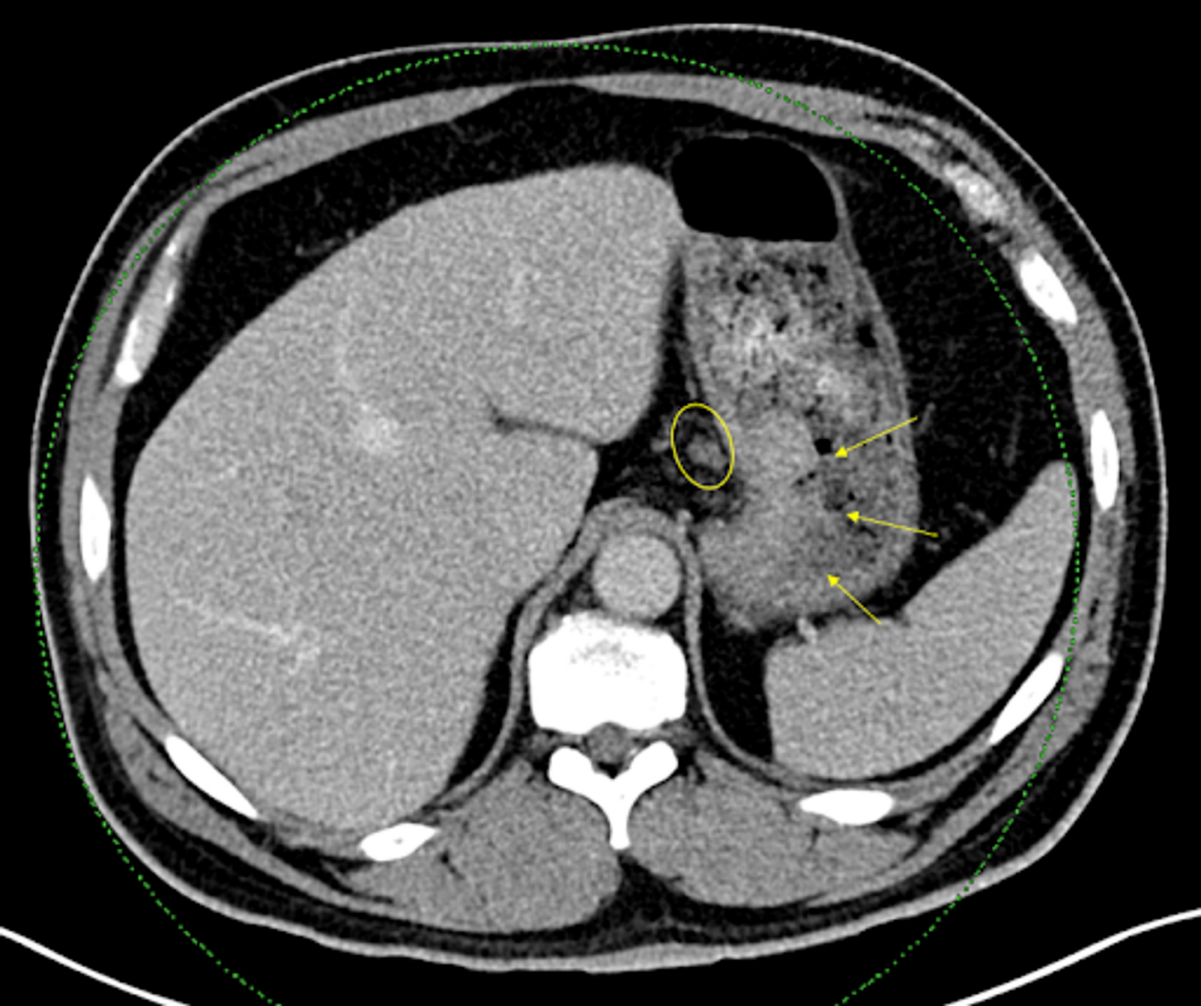
CT scan of the abdomen. The yellow arrows depict a large polypoid soft tissue mass arising from the lesser curvature of the stomach extending posteriorly towards the gastric cardia measuring at least 3.4 cm x 6.0 cm, without extension into distal esophagus or through the wall of the stomach. The yellow circle depicts one prominent peri-gastric lymph node measuring 11 mm, indeterminate for metastatic disease.

A biopsy was taken of the fundal mass, with pathology revealing intramucosal adenocarcinoma with human epidermal growth factor receptor 2 (HER2) immunohistochemical stain positive. Bloodwork detected positive intrinsic factor antibodies. Staging CT scan (Figure 2) showed localized soft tissue mass along the stomach's proximal lesser curvature towards the cardia, in keeping with known adenocarcinoma, and one peri-gastric lymph node. With a multidisciplinary team approach, a decision was made to start the patient on neoadjuvant chemotherapy followed by surgery in three months, given cancer's penetration to the intramucosal layer.

After hospitalization, he followed up at our oncology office, where he was started on neoadjuvant therapy with FOLFOX, which consists of leucovorin calcium (FOL), fluorouracil (F), and oxaliplatin (OX), in addition to dexamethasone. He has received six cycles so far, one cycle every two weeks. He has shown significant improvement in his blood counts, with the most recent complete blood count showing Hgb 13.5 g/dL and MCV 92.6 fL (Table 1).

**Table 1:** Trend of CBC throughout hospitalization and during treatment. CBC, complete blood count; WBC, white blood cell; RBC, red blood cell; Hgb, hemoglobin; Hct, hematocrit; MCV, mean corpuscular volume; MCH, mean corpuscular hemoglobin; MCHC, mean corpuscular hemoglobin concentration; RDW,  red cell distribution width

	Day 1	Day 2	Day 3	Day 5	Day 8	Day 15	Day 30	Day 90
WBC (Thou/uL)	4.4	4.7	4.4	6.4	3.6	5.1	4.8	5.9
RBC (mill/UL)	1.08	1.83	2.02	2.45	2.63	3.29	3.76	4.37
Hgb (g/dL)	4.7	6.8	7.3	8.6	9.1	10.8	11.7	13.5
Hct (%)	13.4	19.7	20.9	25.1	27.1	32.3	34.9	40.5
MCV (fL)	124.2	107.8	103.7	102.3	102.8	98.4	92.8	92.6
MCH (pg)	43.3	37.3	36.3	35.3	34.5	33	31.2	30.8
MCHC (g/dL)	34.9	34.6	35	34.5	33.5	33.5	33.6	33.3
RDW (%)	21.9	34.4	32.2	30.8	29.1	23.1	19.2	20.1
Platelets (Thou/UL)	146	150	119	105	84	353	226	104

Repeat imaging with CT abdomen has shown an excellent response to chemotherapy, and the patient is currently undergoing pre-operative clearance in preparation for surgical resection of the tumor. After surgery, he will be started on trastuzumab given his tumor expressed HER2. 

## Discussion

Vitamin B12, or cobalamin, is found readily in animal products such as eggs, dairy, and meat [2]. Once ingested, it eventually binds to a glycoprotein called intrinsic factor, forming a B12-intrinsic factor complex, which is then readily absorbed in the small intestine ileum. Intrinsic factor is produced by parietal cells in the stomach, which also secrete hydrochloric acid to maintain the highly acidic environment found in the stomach [1]. A majority of the total body stores of vitamin B12 are in the liver, with deficiency taking at least one to two years, if not longer, to develop. Many etiologies behind vitamin B12 deficiency include insufficient dietary intake, medication-induced, i.e., metformin, proton pump inhibitors, gastritis, bariatric surgery, pancreatic insufficiency, and/or genetic disorders [2].

A well-recognized cause of vitamin B12 deficiency is PA, resulting from chronic atrophic autoimmune gastritis. In PA, anti-parietal cell antibodies and anti-intrinsic factor antibodies are prevalent, resulting in atrophic damage to the gastric mucosa [3]. With decreased parietal cells and intrinsic factor, there is decreased vitamin B12 absorption; in addition, parietal cells' loss results in hypochlorhydria. While vitamin B12 supplementation treats the anemia, it does not treat the underlying autoimmune gastritis, or does it restore the stomach's acidity. Therefore, PA has been associated with an increased risk of gastric cancer secondary to the chronic inflammation and hypochlorhydria seen with the condition [4].

Gastric adenocarcinoma is one of the most common cancers worldwide [5], with approximately 22,000 patients diagnosed in the United States annually [6]. Most patients present with weight loss and abdominal pain, with many patients having a known history of gastritis and/or gastric ulcer. Imaging with CT scans along with upper endoscopy with biopsy is recommended for tissue diagnosis and anatomic localization. Once gastric adenocarcinoma is confirmed through biopsy, management is guided by the extent of the spread. In patients with potentially resectable gastric cancer (clinical T2N0 or higher), neoadjuvant therapy prior to surgery is recommended, with multiple chemotherapy regimens available. In patients with tumors expressing HER2, the addition of trastuzumab to therapy has been shown to improve survival and outcomes [7].

## Conclusions

Primary gastric cancer is a fatal malignancy with a poor prognosis as patients remain asymptomatic until late-stage progression. PA is a common risk factor for the development of gastric cancer. Clinicians must have a high index of suspicion in patients with B12 deficiency to obtain proper radiographic imaging along with esophagogastroduodenoscopy (EGD) in cases warranting gastric cancer screening. Early diagnosis and treatment will significantly impact both morbidity and mortality.
